# Investigation of the Relationships Between IL-12B and IL-23 Receptor Polymorphisms with Behçet’s Disease in a Turkish Population

**DOI:** 10.3390/ijms27020923

**Published:** 2026-01-16

**Authors:** Sanem Arıkan, Onur Öztürk, Ayfer Atalay, Erol Ömer Atalay

**Affiliations:** 1 Department of Biophysics, Faculty of Medicine, Pamukkale University, 20160 Denizli, Turkey; 2 Department of Biophysics, Faculty of Medicine, Malatya Turgut Özal University, 44210 Malatya, Turkey

**Keywords:** Behçet’s disease, Interleukin-12B, Interleukin-23R, haplotype, single nucleotide polymorphism

## Abstract

Behçet’s disease is a chronic, multisystem inflammatory disorder characterized by recurrent mucocutaneous and ocular manifestations, in which genetic factors, particularly cytokine gene polymorphisms, are thought to contribute to disease susceptibility. This study aimed to investigate the association of *Interleukin-12B* and *Interleukin-23R* gene polymorphisms and haplotype distributions with Behçet’s disease in Denizli, a province of Turkey. A total of 88 patients with Behçet’s disease and 133 healthy controls were genotyped for *Interleukin-12B* (rs3213119, rs3213120, rs3212227, rs3213113, rs2082412) and *Interleukin-23R* (rs1004819, rs7517847, rs7530511, rs10489629, rs10889677) polymorphisms using polymerase chain reaction–restriction fragment length polymorphism analysis. Genotype, allele, and haplotype distributions were evaluated for associations with Behçet’s disease risk and clinical manifestations. The results demonstrated that the *Interleukin-12B* rs2082412 G allele and rs3213119 G allele were associated with increased risk of Behçet’s disease. Additionally, the *Interleukin-23R* rs7530511 TT genotype and rs10489629 GG genotype and G allele were significantly associated with Behçet’s disease susceptibility. Haplotype analyses revealed AAAGG and GTCAC as the most frequent haplotypes in *Interleukin-12B* and *Interleukin-23R* loci, respectively, in both patients and controls. These findings suggest that *Interleukin-12B* and *Interleukin-23R* gene polymorphisms and haplotypes may be associated with Behçet’s disease susceptibility and clinical heterogeneity in this population.

## 1. Introduction

Behçet’s disease (BD) represents a persistent, multisystemic condition typified by recurring oral ulcers, genital ulcers, and uveitis, affecting various tissues and systems [1]. Studies have shown that immune system mechanisms and their components play a crucial role in developing different inflammatory and autoimmune diseases [2]. Although BD has a worldwide distribution, it is commonly seen in the ‘Silk Road’ region. The geographic position of Turkey, particularly Denizli province, alongside the pivotal role of cytokines in BD, motivated this study to focus on Interleukin (IL)-6, IL-8, IL-17, IL-23, and their respective receptors within this population [3,4,5].

In recent years, T helper 17 (Th17) cell subtype and related cytokines have been studied. The most effective cytokine produced by Th17 cells is IL-17. The stabilization and development of Th17 cells require cytokine IL-23. Consequently, the IL-23/IL-17 pathway has been extensively examined in the context of numerous autoimmune disorders [6]. Studies on cytokine–cellular interactions in Behçet’s disease have demonstrated significant differences in immune cells, such as increased percentages of T cells, natural killer (NK) cells, and monocytes in patients with Behçet’s disease compared to healthy controls [7]. In addition to this, polymorphism studies have shown relationships between the *IL-12B* and *IL-23R* genes and various autoimmune diseases such as multiple sclerosis, psoriasis, rheumatoid arthritis (RA), inflammatory bowel disease, ankylosing spondylitis, sarcoidosis, arthritis and Crohn’s disease [8,9,10]. Increased neutrophilic infiltration in typical BD lesions aligns with the activation of T-helper cells (Th1, Th17), which play a crucial role in BD-associated inflammatory responses [11]. Furthermore, serum levels of cytokines, and chemokines such as IL-1, IL-2, IL-8, IL-12, IL-17, IL-22, tumor necrosis factor-alpha (TNF-α), and interferon-gamma (IFN-γ) or their receptors have been reported in patients with active BD [12,13,14]. Investigations into the IL-23/IL-17 axis in the pathogenesis of BD have demonstrated heightened IL-23 expression in erythema nodosum-like lesions in patients with active BD or uveitis [15,16,17].

The heterodimeric cytokine, IL-23, consists of the subunits IL-12 p40 and IL-23 p19 [18]. The structural similarities with IL-12 and IL-23 exhibit distinct immunological roles. The complex of IL-23 is predominantly released by macrophages and monocyte-derived dendritic cells, playing a pivotal role in the maintenance of Th17 cells and potentially contributing to the formation of Th17 memory cells [19]. Studies have shown that polymorphisms in the *IL-12B* gene are associated with various immune-related disorders, including psoriasis, RA, or certain cancers [20,21,22]. Consequently, the *IL-12B* gene represents a promising candidate for investigating susceptibility to BD. In this study, all of the polymorphic loci for IL-23 are in the gene region of *IL-12B* subunit p40. This polymorphic region may help to clarify the relative contribution of these two cytokines in immune regulation.

The functional receptor, IL-23R, consists of IL-12Rβ1, and IL-23Rβ2 binds to relative ligand IL-23. It is expressed mostly in activated memory T cells as well as in NK cells and innate lymphoid cells [23]. In addition, it has been shown that IL-23R is required for the differentiation of helper T cells for the production of IL-17 [24].

The relationship between BD and granulomatous diseases is complex and shares some common features at the immunopathological level. Ulcerations and ocular involvements are frequently seen in the patient profile. Although BD is not granulomatous, some pathways intersect with granulomatous diseases such as T cell-mediated immune system and cytokine (TNF-α, IL-17, IL-23, etc.) profiles. This study will be of interest to future studies as it provides novel insights into genetic risk factors contributing to granulomatous and/or autoimmune diseases [2,14].

Previously, we explored the effects of haplotypes of SNPs *CXCR1*, *CXCR2*, *CXCL5,* and *CXCL8 (IL-8)* which stimulate neutrophilic migration and activation concerning BD [3,4]. We tried to identify the potentially related haplotypes arising from *IL-12B* and *IL-23R* SNP loci regarding BD. In our study, possible haplotypes were determined by studying the polymorphisms of different loci for *IL-12B* (rs3213119, rs3213120, rs3212227, rs3213113, and rs2082412, respectively) and *IL-23R* (rs1004819, rs7517847, rs7530511, rs10489629 and rs10889677, respectively) which play a crucial role in immune regulation in BD. Therefore, we aimed to investigate the possible contributions of genetic polymorphisms in the neutrophilic cytokine (IL-12B) and its receptor (IL-23R) to BD pathogenesis in the Denizli province of Turkey.

## 2. Results

**Figure 1:**
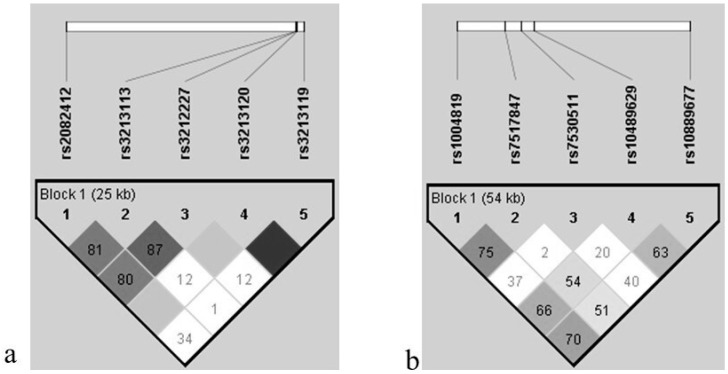
Plot of pairwise linkage disequilibrium (LD) analysis of (**a**) *IL-12B* and (**b**) *IL-23R* standard D’ was shown in the plot. Empty squares indicate a high degree of LD between pairs of markers. Numbers indicate the D’ value expressed as a percentile. Dark gray squares indicate pairs in strong LD with LOD scores for LD ≥ 2; light gray squares, D’ = 1 with LOD = 2; white squares, D’ < 1.0 and LOD ≤ 2.

The genotype and allele frequencies of *IL-12B* and *IL-23R* SNPs in BD and HCs are presented in Table 1 and Table 2, respectively. According to pairwise linkage disequilibrium (LD) analysis of *IL-12B*, strong pairs of BD and HCs are shown in Figure 1a. It is remarkable that the SNPs rs3213120 and rs3213119 associated with *IL-12B* are in complete LD. However, considering the contribution of all SNPs to the population distribution of haplotype data, haplotype analyses were performed including these two SNPs. The genotype distribution of polymorphisms in *IL-12B* showed that SNPs were consistent with the HWE in both groups (*p* > 0.05), except rs2082412, which was not consistent in HCs (*p* = 0.00535).

The frequencies of rs2082412 AA and GG genotypes, A and G alleles, showed statistically significant differences between BD and HCs. Due to the lower frequency of rs3213119 T allele (*p* = 0.0196) (BD; 0.57%, HCs; 6.01%) and the absence of TT genotype in BD individuals, the GG (*p* = 0.02111) and GT (*p* = 0.0260) genotype and allele frequencies in this region were substantially greater in the HCs. Although the frequencies of rs2082412 AA, rs3213119 GG, and GT genotypes initially showed significance, we applied the Bonferroni correction to eliminate potential errors arising from multiple testing; consequently, these specific associations did not remain significant (*p* ^c^ > 0.05), ensuring that our final conclusions are based only on the most robust and statistically validated findings.

BD clinical findings (Supplementary Materials Table S1) with statistical significance of *IL-12B* and *IL-23R* SNPs were summarized in Supplementary Materials Tables S3 and S4. Frequencies of the rs2082412 genotype GG and G allele were significantly higher in the female BD patients compared with the female HCs. However, no statistically significant difference was found in the rs2082412 distribution patterns of genotypes and alleles among male BD patients and male HCs. Frequency distributions of other SNP loci in the *IL-12B* gene are not different between gender groups of BD and HCs.

The frequency of the rs2082412 AA genotype was significantly lower in BD patients with a positive pathergy test (PPT) (*p* = 0.0145). In contrast, BD patients with PPT showed significantly higher frequencies of the rs3212227 A allele and AA genotype compared with those without PPT, while the frequency of the C allele was significantly lower in PPT-positive patients (*p* = 0.0176 and *p* = 0.0485, respectively). Similarly, the rs3213113 A allele was significantly more frequent in PPT-positive patients, whereas the C allele was less frequent compared with PPT-negative patients (*p* = 0.0301). However, the rs3213113 C allele was found to have a higher frequency than the A allele in BD patients with arthritis (*p* = 0.0204). When the Bonferroni correction was applied, the significance was lost only in the rs3212227 AA genotype group of the BD patients with PPT.

*IL-12B* haplotypes and frequencies in BD patients and HCs were estimated using Arlequin v3.5.1.3. Sixteen haplotypes were identified in both groups; GCCAT was exclusive to BD patients, whereas ACAGG, GAAGT, GCCGT, ACCGT, ACAAT, AAAGT, GAAAT, and AAAAT were detected only in HCs (Table 3). AAAGG was the most frequent haplotype in both groups (BD: 40.34 ± 3.71; HCs: 47.37 ± 3.07), while GACGG was the least frequent (BD: 0.57 ± 0.57; HCs: 1.50 ± 0.75). Global differentiation analysis revealed significant differences in haplotype distributions between BD patients and HCs (*p* = 0.00009 ± 0.0001), as well as between HCs and BD patients without PPT (*p* = 0.01880 ± 0.0047), with genital lesions (*p* = 0.00302 ± 0.0015), or with arthritis (*p* = 0.00366 ± 0.0009). Differences were also observed between BD patients with/without PPT (*p* = 0.00778 ± 0.0016), and between PPT-positive patients and those with ocular involvement (*p* = 0.03860 ± 0.0030), without erythema nodosum (*p* = 0.04443 ± 0.0039), and with/without arthritis (*p* = 0.03815 ± 0.0052; *p* = 0.04105 ± 0.0043). No significant differences were detected between HCs and other BD clinical features.

Pairwise linkage disequilibrium (LD) analysis of *IL-23R* revealed strong LD between pairs in BD and HCs, as illustrated in Figure 1b. The distribution of *IL-123R* rs10889677 allele frequencies in both populations (*p* = 0.00996, *p* < 0.0000, respectively), rs1004819 (*p* = 0.03391), and rs7530511 (*p* = 0.00032) in HCs are not consistent with HWE; however, the other SNPs are consistent with HWE in both groups (*p* > 0.05).

When the allele and genotype frequencies of rs10889677, rs1004819, and rs7530511 loci were evaluated within themselves in terms of genetic variation and population structure; the frequencies of *IL-23R* rs1004819 GT, and TT genotypes and rs7530511 TT genotype were found to be statistically significant between BD patients and HCs (*p* = 0.0246, *p* = 0.0435, *p* = 0.0109, respectively). The frequency of rs10889677 AA genotype was lower in BD patients with erythema nodosum than those without this lesion (*p* = 0.0412). The frequency distribution of rs1004819 GG and GT genotypes were found to be significant between BD patients with arthritis and those without arthritis (*p* = 0.0212, *p* = 0.0277). However, the rs7530511 CT genotype frequency is lower in BD patients with ocular lesions than those without this lesion (*p* = 0.0185) (Supplementary Materials Table S4). *IL-23R*, rs10489629 GG genotype (*p* = 0.0035), and G and A allele (*p* = 0.0109) frequencies were found to be significantly elevated in BD patients than HCs (Table 2).

The frequencies of rs7517847 GG genotype (*p* = 0.0177) was found to be higher in BD patients with ocular lesions compared to patients without lesion. Although rs7517847 GG genotype and G allele frequencies were lower in BD patients with genital lesions compared to patients without this lesion (*p* = 0.0294, *p* = 0.0351, respectively), rs7517847 T allele frequency was found to be higher (*p* = 0.0351). When the Bonferroni correction was applied, the significance was lost in rs7517847 G and T allele groups of BD patients with genital lesions.

30 different haplotypes containing the rs1004819, rs7517847, rs7530511, rs10489629, and rs10889677 SNPs in the *IL-23R* gene were found in BD patients and HCs (Table 4) by Arlequin software (ver 3.5.1.3). Haplotypes GTTGA, TTCGC, GGTAC, and TTTGC were detected only in the BD group. The GGCAA, GGTA, and GTCGA haplotypes were found only in the HCs. The haplotype GTCAC has the highest frequency in both groups while the haplotypes TGCAA, GTCAA, TGTGC, TGCGC, GGTGC, and TTTAC were observed to be at the lowest frequency. Global test results indicated that the distribution of BD patients’ haplotype frequencies compared with the HCs was statistically different (*p* = 0.01344 ± 0.0025). Additionally, haplotype frequency distributions were found to be significantly different between HCs and BD patients with and without genital lesions (*p* = 0.01527 + -0.0026 and *p* = 0.00357 ± 0.0011, respectively), erythema nodosum (*p* = 0.00349 ± 0.0014), with papulopustular lesions (*p* = 0.00904 ± 0.0019), with PPT (*p* = 0.01408 ± 0.0042), with and without ocular involvement (*p* = 0.00083 ± 0.0007 and *p* = 0.00453 ± 0.0016, respectively) and with arthritis (*p* = 0.00031 ± 0.0003). The result of the global differentiation test also indicates a statistical difference between BD patients with arthritis and those without this symptom (*p* = 0.01079 ± 0.0024).

Overall, our data indicate that among the analyzed polymorphisms, rs2082412 (*IL-12B*, G allele), rs7530511 (TT genotype), and rs10489629 (*IL-23R*, G allele) were significantly more frequent in BD patients, suggesting their possible association with increased disease risk. In contrast, other variants showed lower frequencies among BD patients, which may indicate potential protective roles.

## 3. Discussion

Behçet’s disease (BD) is a systemic vasculitic disorder with a complex immunopathogenesis characterized by dysregulated inflammatory responses involving multiple organs and tissues, that is influenced by both genetic and environmental factors [1,25]. Investigating the associations between genetic variations in cytokine genes (e.g., *IL1*, *IL6*, *IL8*, *IL10*, *IL17*, *IFN-γ*, *TNF-α*) and the pathogenesis of BD offers valuable insights into disease mechanisms [3,4,26,27].

Within the framework of BD immunopathogenesis, several studies have explored associations between clinical features and serum levels or various cytokine gene polymorphisms [28,29] and both Th1- and Th17-mediated immune responses are central. Elevated IL-12 and IFN-γ levels stimulate Th1 polarization, while increased IL-23 and IL-17 production promotes Th17 activation, sustaining chronic inflammation and neutrophil recruitment to vascular tissues [17]. Chi et al. demonstrated elevated IL-23, IL-17, and IFN-γ levels in BD patients with active uveitis, supporting the involvement of IL-23/IL-17 and IFN-γ pathways in ocular inflammation. Although Baygutalp et al. found no significant difference in IL-23 levels across clinical subgroups, ongoing studies continue to highlight the functional relevance of this axis at both serum and genetic levels [15].

Studies on polymorphisms have highlighted the potential susceptibility of *IL-12B* and *IL-23R* genes to BD. Mizuki et al. identified this in studies of 26 SNPs with Turkish and Korean patients, where only rs1495965 was associated with BD [8]. The polymorphic region, *IL-12B*, rs3212227 is strongly associated with psoriasis and plays a crucial role in disease pathogenesis [30,31,32]. The studies suggested that SNPs such as rs3212227 and rs2082412 were susceptible to BD [33]. *IL-12B*, rs3212227 has also been found in the most relevant region with the BD and the individuals carrying the C allele had a potential risk of developing the disease [34]. While previous studies reported an association of rs3212227 C allele with increased susceptibility to BD, our results indicated higher frequency of A allele and AA genotype with PPT positivity rather than overall disease susceptibility. This may be explained by the phenotypic heterogeneity or possibly reflects the variable activation of Th1/Th17 pathways.

Unlike these results, according to OR, we determined that *IL-12B*, rs2082412 AA genotype, allele A, and rs3213119 GT genotype and allele T may be protective for BD. But Bonferroni correction showed that rs2082412 GG and G allele could be related to a higher risk of BD. Also, the OR and *p*-values showed that the rs3213119 G allele may play a role in the development of BD. Although the Bonferroni correction reduces the number of statistically significant findings, it is important to note that this method can have significant implications for further analysis. The HWE and LD results of *IL-12B* and *IL-23R* suggested that SNPs may have developed at different times in BD and HCs populations.

Following the identification of *IL-23R* polymorphisms in Crohn’s disease, their associations with other inflammatory disorders, including psoriasis, rheumatoid arthritis (RA), and Behçet’s disease (BD), have been extensively investigated [35,36,37]. Genome-wide association studies (GWASs) by Mizuki and Remmers identified *IL-23R* and *IL-10* as susceptibility loci for BD and suggested their potential as therapeutic targets [8,38]. While Yalçın et al. reported no association between BD and *IL-23R* SNPs rs7517847 and rs1004819 [10], Xavier et al. demonstrated a significant association for rs7517847 [39]. Additionally, rs10889677 has been implicated in the etiology of RA [40]. Such discrepancies may be attributed to differences in ethnic background, genetic structure, sample size, and study design. In this context, our findings revealed a significant association only for the *IL-23R* rs7530511 TT genotype and the rs10489629 GG genotype and G allele, suggesting a possible population-specific genetic effect in the Denizli province. Nevertheless, the relatively limited sample size and the single-population design should be considered when interpreting these results.

Haplotype analyses provide additional insight into the complex genetic architecture of BD. Previous studies have demonstrated that functional haplotypes within *IL-12B* or *IL-23R* loci may confer susceptibility or protection in inflammatory conditions such as childhood atopic asthma, Crohn’s disease, and psoriasis, underscoring the relevance of haplotype-based approaches even in the absence of significant single-SNP associations [41,42,43]. In the present study, no statistically significant differences in haplotype distributions were observed between BD patients and healthy controls. Nevertheless, the presence of multiple haplotypes and their varying frequencies reflects considerable polymorphic diversity within the *IL-12B* and *IL-23R* genes in this population. The AAAGG haplotype was the most frequent among *IL-12B* variants in both groups, while GTCAC was the most common haplotype within the *IL-23R* locus (Table 4). Moreover, combined analysis of *IL-12B* and *IL-23R* loci revealed a marked increase in haplotype diversity (Supplementary Materials Table S5). Consistent with previous findings involving *IL-8* and *CXCR1/CXCR2* receptor gene polymorphisms in BD, this extensive diversity supports the notion that cytokine and cytokine receptor gene polymorphisms exhibit substantial heterogeneity and may collectively contribute to BD pathogenesis [3,4].

When evaluating clinical findings across the same patient cohort analyzed for different chemokine/cytokine and receptor SNPs, distinct associations between haplotypes and specific clinical manifestations of BD became evident. Previous studies in this cohort demonstrated differences in *IL-8* haplotypes among patients with arthritis, genital ulcers, ocular involvement, papulopustular lesions, erythema nodosum, and pathergy positivity [3]. In addition, *CXCR1* haplotypes differed between patients with arthritis and erythema nodosum compared to controls, while *CXCR2* haplotypes were significantly associated with genital and ocular involvement [4]. In the present study, integration of genetic and clinical data revealed that *IL-12B* haplotypes were associated with genital ulcers and arthritis, whereas *IL-23R* haplotypes correlated with erythema nodosum, pathergy positivity, ocular involvement, and papulopustular lesions (Supplementary Materials Table S6). These findings support a contributory role of *IL-12B* and *IL-23R* polymorphisms in BD susceptibility. Notably, higher frequencies of rs2082412 (*IL-12B*, G allele), rs7530511 (*IL-23R*, TT genotype), and rs10489629 (*IL-23R*, G allele) in BD patients suggest a potential involvement in disease pathogenesis through modulation of cytokine signaling pathways, whereas lower frequencies of other variants may indicate protective effects. Our findings highlight the potential functional impact of specific *IL-12B* and *IL-23R* variants in the pathogenesis of Behçet’s Disease (BD). Specifically, the *IL-23R* rs7530511 variant is a missense mutation located in exon 8 that can alter the extracellular domain of the receptor and its affinity for IL-23. Furthermore, the association of 3′ UTR variants such as *IL-12B* rs3212227 and *IL-23R* rs10889677 suggests a regulatory role in mRNA stability and microRNA binding. The high evolutionary conservation of these loci further supports their critical roles in modulating inflammatory responses. These results underscore the importance of genetic variations in key cytokine pathways as susceptibility factors for BD in the Turkish population and we believe they may provide a foundation for future studies.

Collectively, these variants may influence Th1/Th17-mediated immune responses, thereby contributing to inflammatory susceptibility and clinical heterogeneity in Behçet’s disease. This underscores the importance of haplotype-based genetic analyses in linking molecular variation with clinical phenotypes. However, several limitations should be considered. First, the study was conducted in a dermatology clinic, which may have led to underrepresentation of patients with predominantly non-dermatological manifestations. Second, the absence of functional analyses and disease severity assessments limits interpretation of whether the observed polymorphisms have direct pathogenic relevance or represent associative findings. Finally, as the study population was restricted to a single geographic region of Turkey (Denizli), some associations may reflect regional or ethnic genetic characteristics rather than disease-specific effects.

In the present study, the estimated prevalence of BD in the study population was approximately 8 per 100,000, which is lower than the prevalence reported for Turkey by Yurdakul et al. [44]. As this represents the first investigation of *IL-12B* and *IL-23R* gene polymorphisms in relation to BD in the Denizli region, the sample size was considered adequate for an exploratory genetic association study. Nevertheless, larger multi-center studies incorporating diverse clinical settings and functional assays are required to validate these findings and to further elucidate the mechanistic impact of these polymorphisms on cytokine signaling and clinical outcomes.

## 4. Materials and Methods

### 4.1. Patients and Controls

This study includes 88 individuals diagnosed with BD (48 male and 40 female) and 133 healthy individuals (HCs) (66 male and 67 female). The BD patients were followed at the Department of Dermatology, Faculty of Medicine, Pamukkale University, and were diagnosed in accordance with the International Study Group classification [45]. The clinical features of patients are summarized in Supplementary Materials Table S1. Cases with vascular, neurological, or gastrointestinal involvements were removed from the study due to their occurrence in fewer than ten patients. Controls were selected from individuals with no autoimmune disease or allergy. Written informed consent has already been taken from BD patients and HC individuals for further DNA analysis. DNA samples were collected from an anonymous DNA bank established by the framework of the projects. Ethical approval was obtained from the Ethics Committee of Pamukkale University, Denizli, Turkey. (Ethical Approval No.: 2018/04/21, 20 February 2018, Denizli, TURKEY). This study was also supported by PAU-ADEP/2017 Researcher Project No.: 2017KRM002-303.

### 4.2. Genotyping

Genomic DNA was extracted by standard blood/phenol–chloroform method from blood samples [3]. Genotypes of *IL-12B* (OMIM: 161561) [rs3213119 (C > A), rs3213120 (C > T), rs3212227 (T > A, G), rs3213113 (T > G), rs2082412 (G > A, T)] *IL-23R* (OMIM: 607562) [rs1004819 (G > A), rs7517847 (T > G), rs7530511 (T > A, C), rs10489629 (T > C), rs10889677 (C > A)] were investigated by polymerase chain reaction with restriction fragment length polymorphism (PCR-RFLP). All PCR reactions were performed in a final volume of 50 μL and were carried out using Taq DNA polymerase (Thermo Fisher Scientific, Waltham, MA, USA) and 200 μM of deoxyribonucleotide triphosphate (dNTP) (Thermo Fisher Scientific, Waltham, MA, USA), 0.1 μM primers (Oligomer Biotechnology, Ankara, Turkey), and 2 mM MgCl_2_ (Thermo Fisher Scientific, Waltham, MA, USA). The general PCR thermal profile consisted of an initial denaturation at 95 °C for 5 min, followed by 30–35 cycles of denaturation at 94 °C for 45 s, annealing for 45 s, and extension at 72 °C for 45 s, with a final extension at 72 °C for 7 min. Due to the involvement of ten different loci, the specific PCR- RFLP conditions optimized for each SNP are detailed in Supplementary Table S2 to ensure clarity and precision (Supplementary Materials Table S2). PCR and RFLP designs followed previously published reports [35,46,47]. Representative agarose gel electrophoresis images for the studied SNPs are shown in Supplementary Materials (Figures S1 and S2).

### 4.3. Statistical Analysis

The distribution of allele and genotype frequencies between BD patients and HCs was evaluated using Odds Ratios (ORs) and 95% confidence intervals (CIs) to determine the strength of associations. *p*-values were derived from z-scores using MedCalc software (Version 23.4.5) [48]. Corrections for multiple comparisons were performed using the Bonferroni method, adjusting the *p*-value by the number of genotypes or alleles analyzed for each polymorphic region. Corrected *p*-values (*p* ^c^) below 0.05 were deemed statistically significant. Hardy–Weinberg equilibrium (HWE) was assessed using Arlequin software (Version 3.5.1.3; http://cmpg.unibe.ch/software/arlequin35/, accessed on 10 June 2024) [49]. Haplotype identification was conducted using the maximum likelihood method, with global differentiation testing between patient and control groups performed in Arlequin. Linkage disequilibrium (LD) pairwise values were visualized using Haploview (Version 4.2) [50].

## 5. Conclusions

In conclusion, this study provides evidence supporting an association between *IL-12B* and *IL-23R* gene polymorphisms and haplotypes and susceptibility to Behçet’s disease in a Turkish population. The observed associations of rs2082412 (*IL-12B*) and rs7530511 and rs10489629 (*IL-23R*) with increased disease risk are consistent with previous reports implicating the IL-23/IL-17 axis in the pathogenesis of BD. Moreover, haplotype-based analyses underscore the genetic complexity and clinical heterogeneity of the disease. Although further validation in larger and ethnically diverse cohorts is required, our findings contribute to the growing body of evidence on cytokine-related genetic factors in BD and may support future disease–gene association studies and meta-analyses.

## Figures and Tables

**Table 1:** Genotype and allele frequencies of the *IL-12B* SNPs.

	HCs %	BD %	OR	95% CI	*p* ^a^	*p* ^c^
rs2082412						
AA	33.83	19.32	0.47	0.25–0.89	0.0201	ns
AG	57.90	60.23	1.10	0.64–1.91	0.7302	ns
GG	8.27	20.45	2.85	1.27–6.38	0.0108	0.0324
A	62.78	49.43	0.58	0.39–0.85	0.0056	0.0112
G	37.22	50.57	1.73	1.17–2.54	0.0056	0.0112
rs3213113						
AA	57.14	50.00	0.7500	0.4367–1.2882	0.2972	-
AC	36.84	43.18	1.3029	0.7520–2.2573	0.3454	-
CC	6.01	6.82	1.1433	0.3826–3.4160	0.8105	-
A	75.56	71.60	0.8149	0.5297–1.2537	0.3517	-
C	24.44	28.40	1.2271	0.7977–1.8878	0.3517	-
rs3212227						
AA	56.39	51.14	0.8093	0.4714–1.3895	0.4429	-
AC	40.60	42.04	1.0614	0.6144–1.8336	0.8309	-
CC	3.01	6.82	2.3598	0.6463–8.6164	0.1938	-
A	76.69	72.16	0.7877	0.5097–1.2173	0.2826	-
C	23.31	27.84	1.2695	0.8215–1.9618	0.2826	-
rs3213120						
AA	0	0	1.5085	0.0297–76.7302	0.8375	-
AG	2.26	1.14	0.4981	0.0510–4.8669	0.5490	-
GG	97.74	98.86	2.0077	0.2055–19.6176	0.5490	-
A	1.13	0.57	0.5010	0.0517–4.8551	0.5508	-
G	98.87	99.43	1.9962	0.2060–19.3467	0.5508	-
rs3213119						
GG	88.72	98.86	11.0593	1.4335–85.3204	0.0211	ns
GT	10.53	1.14	0.0977	0.0126–07571	0.0260	ns
TT	0.75	0	0.4991	0.0201–12.3904	0.6715	-
G	93.99	99.43	11.2000	1.4716–85.2383	0.0196	0.0392
T	6.01	0.57	0.0893	0.0117–0.6795	0.0196	0.0392

*p* ^a^: *p*-value, *p* ^c^: *p*-value after Bonferroni correction, ns: not statistically significant.

**Table 2:** Genotype and allele frequencies of the *IL-23R* SNPs.

	HCs %	BD %	OR	95% CI	*p* ^a^	*p* ^c^
rs1004819						
GG	24.06	26.14	1.25	0.68–2.31	0.4698	-
GT	60.90	45.45	0.53	0.31–0.92	0.0246	ns
TT	15.04	28.41	2.00	1.02–3.92	0.0435	ns
G	54.51	48.86	0.87	0.60–1.28	0.4865	-
T	45.49	51.14	1.15	0.78–1.68	0.4865	-
rs7517847						
GG	13.03	11.36	1.07	0.46–2.50	0.8661	-
GT	49.62	43.19	0.77	0.45–1.33	0.3480	-
TT	38.35	45.45	1.34	0.78–2.31	0.2937	-
G	36.84	32.95	0.84	0.56–1.26	0.4027	-
T	63.16	67.05	1.19	0.79–1.77	0.4027	-
rs7530511						
CC	42.86	40.91	0.92	0.53–1.59	0.7740	-
CT	54.89	47.73	0.75	0.44–1.29	0.2974	-
TT	2.25	11.36	5.56	1.48–20.80	0.0109	0.0327
C	70.30	64.77	0.78	0.52–1.17	0.2227	-
T	29.70	35.23	1.29	0.86–1.93	0.2227	-
rs10489629						
AA	40.60	32.96	0.72	0.41–1.26	0.2512	-
AG	51.13	44.31	0.76	0.44–1.31	0.3218	-
GG	8.27	22.73	3.26	1.48–7.21	0.0035	0.0105
A	66.20	55.11	0.63	0.43–0.93	0.0196	0.0392
G	33.80	44.89	1.59	1.08–2.35	0.0196	0.0392
rs10889677						
AA	12.78	15.91	1.29	0.60–2.77	0.5130	-
AC	72.93	63.64	0.65	0.36–1.16	0.1439	-
CC	14.29	20.45	1.55	0.76–3.14	0.2313	-
A	49.25	47.73	0.94	0.64–1.38	0.7542	-
C	50.75	52.27	1.06	0.73–1.56	0.7542	-

*p* ^a^: *p*-value, *p* ^c^: *p*-value after Bonferroni correction, ns: not statistically significant.

**Table 3:** Haplotypes and frequencies of *IL-12B*.

	Haplotypes	HCs (% ± sd)	BD (% ± sd)	OR	95% CI	*p* ^a^
1	AAAGG	47.37 ± 3.07	40.34 ± 3.71	0.7518	0.4292–1.3169	0.3185
2	GAAGG	20.68 ± 2.49	30.68 ±3.49	1.6901	0.8901–3.2092	0.1087
3	GCCGG	9.40 ± 1.79	17.05 ± 2.84	2.0710	0.8755–4.8989	0.0975
4	ACCGG	8.27 ± 1.69	9.66 ±2.23	1.2778	0.4824–3.3844	0.6218
5	GCAGG	1.50 ± 0.75	1.14 ± 1.00	0.4949	0.0442–5.5478	0.5684
6	GACGG	1.50 ± 0.75	0.57 ± 0.57	0.4949	0.0442–5.5478	0.5684
7	GCCAT	-	0.57 ± 0.57	3.0302	0.1220–75.2834	0.4988
8	ACAGG	3.01 ± 1.05	-	0.1386	0.0071–2.7185	0.1931
9	GAAGT	2.26 ± 0.91	-	0.1960	0.0093–4.1353	0.2949
10	AACGG	2.26 ± 0.91	-	0.1960	0.0093–4.1353	0.2949
11	GCCGT	1.88 ± 0.83	-	0.1960	0.0093–4.1353	0.2949
12	ACCGT	0.38 ± 0.38	-	1.0000	0.0196–50.8937	1.0000
13	ACAAT	0.38 ± 0.38	-	1.0000	0.0196–50.8937	1.0000
14	AAAGT	0.38 ± 0.38	-	1.0000	0.0196–50.8937	1.0000
15	GAAAT	0.38 ± 0.38	-	1.0000	0.0196–50.8937	1.0000
16	AAAAT	0.38 ± 0.38	-	1.0000	0.0196–50.8937	1.0000

*p* ^a^: *p*-value.

**Table 4:** Haplotypes and frequencies of *IL-23R*.

	Haplotypes	HCs (% ± sd)	BD (% ± sd)	OR	95% CI	*p* ^a^
1	GTCAC	32.33 ± 2.87	21.02 ± 3.08	0.5649	0.2982–1.0700	0.0797
2	TTCAA	10.15 ± 1.85	12.50 ± 2.50	1.3448	0.5603–3.2277	0.5072
3	TGTGA	7.52 ± 1.62	9.66 ± 2.23	1.2778	0.4824–3.3844	0.6218
4	GTCGC	1.50 ± 0.75	7.39 ± 2.00	3.6882	0.7469–18.2112	0.1092
5	TTTAA	6.01 ± 1.46	5.11 ± 1.67	0.8246	0.2433–2.7947	0.7567
6	GGCGC	4.51 ± 1.27	5.11 ± 1.67	1.0000	0.2803–3.5673	1.0000
7	TTCAC	3.01 ± 1.05	4.55 ± 1.57	1.7018	0.3956–7.3209	0.4751
8	TGCGA	7.14 ± 1.58	4.55 ± 1.57	0.6992	0.2143–2.2819	0.5533
9	GGTGA	2.63 ± 0.98	4.00 ± 1.48	1.3472	0.2937–6.1804	0.7014
10	TTTGA	1.13 ± 0.65	4.00 ± 1.48	4.1250	0.4529–37.5744	0.2087
11	TTCGA	3.01 ± 1.05	2.84 ± 1.26	1.0000	0.1969–5.0779	1.0000
12	GTTAA	0.75 ± 0.53	2.27 ± 1.13	2.0204	0.1803–22.6462	0.5684
13	TGTAA	4.51 ± 1.27	2.27 ± 1.13	0.3878	0.0734–2.0474	0.2645
14	GTTAC	1.88 ± 0.83	2.27 ± 1.13	1.0000	0.1381–7.2420	1.0000
15	GGCAC	3.01 ± 1.05	2.27 ± 1.13	0.6599	0.1079–4.0366	0.6528
16	GTTGA	-	1.70 ± 0.98	5.1015	0.2418–107.6245	0.2949
17	TTCGC	-	1.70 ± 0.98	5.1015	0.2418–107.6245	0.2949
18	GTTGC	0.75 ± 0.53	1.14 ± 0.80	1.0000	0.0617–16.2132	1.0000
19	GGCGA	1.50 ± 0.75	1.14 ± 0.80	0.4949	0.0442–5.5478	0.5684
20	GGTAC	-	1.14 ± 0.80	3.0302	0.1220–75.2834	0.4988
21	TGCAA	0.75 ± 0.53	0.57 ± 0.57	1.0000	0.0617–16.2132	1.0000
22	GTCAA	1.88 ± 0.83	0.57 ± 0.57	0.4949	0.0442–5.5478	0.5684
23	TTTGC	-	0.57 ± 0.57	3.0302	0.1220–75.2834	0.4988
24	TGTGC	0.75 ± 0.53	0.57 ± 0.57	1.0000	0.0617–16.2132	1.0000
25	TGCGC	0.38 ± 0.38	0.57 ± 0.57	3.0302	0.1220–75.2834	0.4988
26	GGTGC	2.63 ± 0.98	0.57 ± 0.57	0.3266	0.0334–3.1946	0.3362
27	TTTAC	0.38 ± 0.38	0.57 ± 0.57	3.0302	0.1220–75.2834	0.4988
28	GGCAA	0.75 ± 0.53	-	0.3300	0.0133–8.1992	0.4988
29	GGTAA	0.75 ± 0.53	-	0.3300	0.0133–8.1992	0.4988
30	GTCGA	0.38 ± 0.38	-	1.0000	0.0196–50.8937	1.0000

*p* ^a^: *p*-value.

## Data Availability

The original contributions presented in this study are included in the article/Supplementary Materials. Further inquiries can be directed to the corresponding author.

## Supplementary Materials

The following supporting information can be downloaded at https://www.mdpi.com/article/10.3390/ijms27020923/s1.

## References

[1] Dalvi S.R., Yıldırım R., Yazıcı Y. (2012). Behçet’s syndrome. Drugs.

[2] Akman A., Alpsoy E. (2009). Behcet Hastalığı Etyopatogenezde Guncel Bilgiler. Turkderm.

[3] Atalay A., Arıkan S., Ozturk O., Öncü M., Taşlı M.L., Duygulu Ş., Atalay E.Ö. (2016). The IL-8 Gene Polymorphisms in Behçet’s Disease Observed in Denizli Province of Turkey. Immunol. Investig..

[4] Arıkan S., Atalay A., Öztürk Ö., Duygulu Ş., Atalay E.Ö. (2021). Association of Single Nucleotide Polymorphisms in the CXCR1, CXCR2 and CXCL5 with the Behçet’s Disease in Denizli Province of Turkey. Clin. Exp. Dermatol..

[5] Arıkan S., Öztürk O., Duygulu Ş., Atalay A., Atalay E.Ö. (2023). Associations of IL-17 and IL-17 Receptor Polymorphisms with Behçet’s Disease in Denizli, Province of Turkey. Immunol. Res..

[6] Emmi G., Becatti M., Bettiol A., Hatemi G., Prisco D., Fiorillo C. (2019). Behçet’s Syndrome as a Model of Thrombo-Inflammation: The Role of Neutrophils. Front. Immunol..

[7] Yu Q., Zhang Y., Wu Y., Li M., Pan T. (2025). Identification of immune-associated genes and single-cell sequencing analysis in diagnosing Behçet’s disease. Sci. Rep..

[8] Mizuki N., Meguro A., Ota M., Ohno S., Shiota T., Kawagoe T., Ito N., Kera J., Okada E., Yatsu K. (2010). Genome-wide association studies identify IL23R-IL12RB2 and IL10 as Behçet’s disease susceptibility loci. Nat. Genet..

[9] Wen X., Chen S., Li P., Li J., Wu Z., Li Y., Li L., Yuan H., Tian X., Zhang F. (2017). Single nucleotide polymorphisms of IL12B are associated with Takayasu arteritis in Chinese Han population. Rheumatol. Int..

[10] Yalçın B., Atakan N., Dogan S. (2014). Association of interleukin-23 receptor gene polymorphism with Behçet disease. Clin. Exp. Dermatol..

[11] Direskeneli H. (2001). Behçet’s Disease: Infectius aetiology, new autoantigens, and HLA-B51. Ann. Rheumatol. Dis..

[12] Hamzaoui K., Hamzaoui A., Guemira F., Bessioud M., Hamza M., Ayed K. (2002). Cytokine profile in Behçet’s disease patients. Scand. J. Rheumatol..

[13] Akkurt Z.M., Bozkurt M., Uçmak D., Yüksel H., Uçak H., Sula B., Özkurt Z.G., Yildiz M., Akdeniz D., Arica M. (2015). Serum Cytokine Levels in Behcet’s Disease. J. Clin. Lab. Anal..

[14] Alpsoy E., Bozca B.C., Bilgic A. (2021). Behçet’s Disease: An update for Dermatologists. Am. J. Clin. Dermatol..

[15] Baygutalp F., Altas E.U., Baygutalp N.K., Ozturk N., Sefeoglu B., Melikoglu M.A., Ugur M., Bakan E. (2016). Serum Interleukin-23 Levels and Relationship with Clinical Symptoms of Behçet’s Disease. Eur. Int. J. Sci. Tech..

[16] Lew W., Chang J.Y., Jung J.Y., Bang D. (2008). Increased expression of interleukin-23 p19 mRNA in erythema nodosum-like lesions of Behçet’s disease. Br. J. Dermatol..

[17] Chi W., Zhu X., Yang P., Liu X., Lin X., Zhou H., Huang X., Kijlstra A. (2008). Upregulated IL-23 and IL-17 in Behçet Patients with Active Uveitis. Investig. Ophthalmol. Vis. Sci..

[18] Lupardus P.J., Garcia K.C. (2008). The stucture of Interleukin-23 reveals the molecular basis of p40 subunit sharing with IL-12. J. Mol. Biol..

[19] Tindall E.A., Hayes V.M. (2010). Comprehensive Sequence Analysis of the Human IL23A Gene Defines New Variation Content and High Rate of Evolutionary Conservation. DNA Res..

[20] Torii K., Morita A. (2020). Specific single nucleotide polymorphism genotypes and association of an IL-12B polymorphism with secondary failure of infliximab therapy in Japanese psoriasis patients. J. Dermatol. Sci..

[21] Manolova I., Ivanova M., Vasilev G., Vasilev G., Stoilov R., Miteva L., Stanilova S. (2020). Impact of IL12B Polymorphisms on Genetic Susceptibility and IL-12p40 and IL-23 Serum Levels in Rheumatoid Arthritis. Immun. Investig..

[22] Karimi-Zarchi M., Abbasi H., Javaheri A., Hadadan A., Meibodi B., Tabatabaei R.S., Ghelmani Y., Neamatzadeh H. (2020). Association of IL-12B rs3212227 and IL-6 rs1800795 Polymorphisms with Susceptibility to Cervical Cancer: A Systematic Review and Meta-Analysis. Asian Pac. J. Cancer Prev..

[23] Pepple K.L., Lin P. (2018). Targeting Interleukin-23 in the Treatment of Noninfectious Uveitis. Am. Acad. Ophthalmol..

[24] Jiang Z., Hennein L., Tao Y., Tao L. (2015). Interleukin-23 Receptor Gene Polymorphism May Enhance Expression of the IL-23 Receptor, IL-17, TNF-α and IL-6 in Behcet’s Disease. PLoS ONE.

[25] Saadoun D., Wechsler B. (2012). Behçet’s disease. Orphanet J. Rare Dis..

[26] Morton L.T., Situnayake D., Wallace G.R. (2016). Genetics of Behçet’s disease. Curr. Opin. Rheumatol..

[27] Nanke Y., Yago T., Kotake S. (2017). The Role of Th17 Cells in the Pathogenesis of Behcet’s Disease. J. Clin. Med..

[28] Kim E.S., Kim S.W., Moon C.M., Park J.J., Kim T.I., Kim W.H., Cheon J.H. (2012). Interactions between IL17A, IL23R, and STAT4 polymorphisms confer susceptibility to intestinal Behcet’s disease in Korean population. Life Sci..

[29] Tong B., Liu X., Xiao J., Su G. (2019). Immunopathogenesis of Behcet’s Disease. Front. Immunol..

[30] Cargill M., Schrodi S.J., Chang M. (2007). A Large-scale Genetic Association Study Confirms IL12B and Leads to the Identification of IL23R as Psoriasis-Risk Genes. Am. J. Hum. Genet..

[31] Catanoso M.G., Boiardi L., Macchioni P., Garagnani P., Sazzini M., de Fanti S., Farnetti E., Casali B., Chiarolanza I., Nicoli D. (2013). IL-23A, IL-23R, IL-17A and IL-17R polymorphisms in different psoriatic arthritis clinical manifestations in the northern Italian population. Rheumatol. Int..

[32] Indhumathi S., Rajappa M., Chandrashekar L., Ananthanarayanan P.H., Thappa D.M., Negi V.S. (2016). Investigation of association of the IL-12B and IL-23R genetic variations with psoriatic risk in a South Indian Tamil cohort. Hum. Immunol..

[33] Li X., Bai L., Fang J., Hou S., Zhou Q., Yu H., Kijlstra A., Yang P. (2014). Genetic Variations of IL-12B, IL-12Rb1, IL-12Rb2 in Behcet’s Disease and VKH Syndrome. PLoS ONE.

[34] Xu Y., Zhou K., Yang Z., Li F., Wang Z., Xu F., He C. (2016). Association of cytokine gene polymorphisms (IL-6, IL-12B, IL-18) with Behcet’s disease. Z. Rheumatol..

[35] Eiris N., Santos-Juanes J., Coto-Segura P., Gomez J., Alvarez V., Morales B., Queiro R., Diaz M., Corao A.I., Lopez-Corte K. (2012). Resequencing of the IL12B gene in psoriasis patients with the rs6887695/rs3212227 risk genotypes. Cytokine.

[36] Lu Y., Kane S., Chen H., Leon A., Levin E., Nguyen T., Debbaneh M., Millsop J.W., Gupta R., Huynh M. (2013). The role of 39 Psoriasis Risk Variants on Age of Psoriasis Onset. ISRN Dermatol..

[37] Nititham J., Gupta R., Zeng X., Hartogensis W., Nixon D.F., Deeks S.G., Hecht F.M., Liao W. (2018). Psoriasis risk SNPs and their associations with HIV-1 control. Hum. Immunol..

[38] Remmers E.F., Cosan F., Kirino Y., Ombrella M.J., Abaci N., Satorius C., Le J.M., Yang B., Korman B.D., Cakiris A. (2010). Genome-wide association study identifies variants in the MHC class I, IL10, and IL23R/IL12RB2 regions associated with Behçet’s disease. Nat. Genet..

[39] Xavier J., Shahram F., Davatchi F., Rosa A., Crespo J., Abdollahi B.S., Nadji A., Jesus G., Barcelos F., Patto J.V. (2012). Association Study of IL10 and IL23R–IL12RB2 in Iranian Patients with Behçet’s Disease. Arthritis Rheum..

[40] da Silva I.I.F.G., Angelo H.D., Rushansky E., Mariano M.H., Maia M.M.D., de Souza P.R.E. (2017). Interleukin (IL)-23 Receptor, IL-17A and IL-17F Gene Polymorphisms in Brazilian Patients with Rheumatoid Arthritis. Arch. Immunol. Ther. Exp..

[41] Hirota T., Suzuki Y., Hasegawa K., Obara K., Matsuda A., Akahoshi M., Nakashima K., Cheng L., Takahashi N., Shimizu M. (2005). Functional haplotypes of IL-12B are associated with childhood atopic asthma. J. Allergy Clin. Immunol..

[42] Ferguson L.R., Han D.Y., Fraser A.G., Huebner C., Lam W.J., Morgan A.R. (2010). IL23R and IL12B SNPs and Haplotypes Strongly Associate with Crohn’s Disease Risk in a New Zeland Population. Gastroenterol. Res. Pract..

[43] Oka A., Mabuchi T., Ikeda S., Terui T., Haida Y., Ozawa A., Yatsu K., Kulski J.K., Inoko H. (2013). IL12B and IL23R gene SNPs in Japaneese psoriasis. Immunogenetics.

[44] Yurdakul S., Yazici H. (2008). Behçet’s syndrome. Best. Pract. Res. Clin. Rheumatol..

[45] International Study Group for Behçet’s Disease (1990). Criteria for diagnosis Behçet’s disease. Lancet.

[46] Safrany E., Szell M., Csongei V., Jaromi L., Sipeky C., Szabo T., Kemeny L., Nagy J., Melegh B. (2011). Polymorphisms of the IL23R Gene Are Associated with Psoriasis but not with Immunoglobulin A Nephropathy in a Hungarian Population. Inflammation.

[47] Liu M., Hu X., Wang Y., Chen X., Wu J. (2014). Association of IL-23 and its receptor gene single-nucleotide polymorphisms with multiple sclerosis in Chinese southern population. Int. J. Neurosci..

[48] MedCalc Software. Version 23.4.5. https://www.medcalc.org/calc/odds_ratio.php.

[49] Excoffier L., Laval G., Schneider S. (2005). Arlequin (ver 3.0): An Integrated Software Package for Population Genetics Data Analysis. Evol. Bioinform..

[50] https://www.broadinstitute.org/haploview/haploview.

