# Supplementary material for: Investigation of the Relationships Between IL-12B and IL-23 Receptor Polymorphisms with Behçet’s Disease in a Turkish Population
Source: Int J Mol Sci. 2026 Jan 16;27(2):923. doi: 10.3390/ijms27020923 (PMC12841561; doi:10.3390/ijms27020923)
Supplement: Supplementary file 1 [file ijms-27-00923-s001.zip › ijms-4087096-supplementary.pdf]

Supp Table S1. Clinical features of BD patients (n=88)

**CLINICAL FEATURES**	**BD (%)**
Male (M)	48 (55)
Female (F)	40 (45)
Oral ulcer (OU)	88 (100)
Genital ulcer (GU)	69 (78)
Erythema nodosum (EN)	37 (42)
Papulopustular lesions (PL)	55 (63)
Positive Pathergy test (PPT)	36 (41)
Ocular involvement (OI)	41 (47)
Arthritis (A)	53 (60)

Supp Table S2. SNPs and PCR-RFLP conditions

	SNP	Primers	RE	Denaturation	Annealing	Extension	Cycle number	PCR products	RFLP
**IL-12B**	rs3213119 (G/T)	5'-ggCTTgTTTTgggAgAgTATgCATT-3'	Tai I	94°C /30 sec.	63°C /45 sec.	72°C /1 min	35 cycle	360 bp	G:360 bp
		5'-gTTTCTgATTCTggCAACTgggTg-3'							T:226+134 bp
	rs3213120 (G/A)	5'-CACATCAACTTTTggCATTCTCTTCCC-3'	Hinf I	94°C /1 min	63°C / 45 sec.	72°C /1 min	30 cycle	251 bp	G: 251 bp
		5'-gCAACTTgAgAgCTggAAAATCTATAC-3'							A: 223+28 bp
	rs3212227 (A/C)	5'-TTCTATCTgATTTgCTTTA-3'	Taq I	94°C /30 sec.	43°C / 45sec.	72°C /1 min	30 cycle	233 bp	A: 233 bp
		5'-TgAAACATTCCATACATCC-3'							C: 165+68 bp
	rs3213113 (G/T)	5'-TTTggAggAAAAgTggAAgA-3'	Taq I	94°C /1 min	55°C / 45 sec.	72°C /1 min	40 cycle	300 bp	A: 300 bp
		5'-AACATTCCATACATCCTggC-3'							C: 204+96 bp
	rs2082412 (A/G)	5'-ATggACAACCAAgAAATAgTCATTTACAgC-3'	Hha I	94°C /1 min	64°C / 1 min	72°C /1 min	35 cycle	169 bp	A: 169 bp
		5'-AAAgATCAgATTgCAACAAAgCACCTTTg-3'							G: 139+30 bp
**IL-23R**	rs1004819 (C/T)	5'-ATCTggTggAAATATgTgAAACCTA-3'	Taa I	94°C /1 min	54°C / 45 sec.	72°C /1 min	30 cycle	270 bp	C:13+71+185bp
		5'- gCATTCTAggACCgTTTTgg-3'							T: 13+257 bp
	rs7517847 (T/G)	5'- AAACATTgACATTCCCTTCATAC-3'	BseMI I	94°C /45 sec.	55°C / 1 min	72°C /1 min	35 cycle	530 bp	T: 29+91+410 bp
		5'- gAAATgAgTCACCAATAATCCAC-3'							G: 29+501 bp
	rs7530511 (C/T)	5'- TACCCATCCATTTTAggTTAAAgAA-3'	Hph I	94°C /1 min	60°C / 45 sec.	72°C /1 min	35 cycle	614 bp	C: 51+134+429 bp
		5'-gTCTTgAAgTCCTgACCTAAggTAATC-3'							T: 185+429 bp
	rs10489629 (A/G)	5'-TATAAgCTTgTTTgATTATgATgTCAgCAA-3'	Ssp I	94°C /1 min	55°C / 45 sec.	72°C /1 min	35 cycle	348 bp	A: 31+119+198 bp
		5'- CCACACCTCgCCAAgACTTT-3'							G: 150+198 bp
	rs10889677 (C/A)	5'- ATC gTg AAT gAg gAg TTg CC -3'	Mnl I	94°C /1 min	64°C / 45 sec.	72°C /1 min	30 cycle	470 bp	C: 61+185+224 bp
		5' - TgT gCC TgT ATg TgT gAC CA -3'							A: 185+285 bp

Supp Table S3. The distribution of *IL-12B* gene polymorphisms in gender and clinical findings of BD patients

Sex	n	rs3213119					rs321320					rs3212227					rs3213113					rs2082412				
		GG	GT	TT	G+	T-	AA	AG	GG	A+	G-	AA	AC	CC	A-	C+	AA	AC	CC	A-	C+	AA	AG	GG	A-	G+
C-M	66	1	6	59	8	124	0	1	65	1	131	35	29	2	99	33	37	27	2	101	31	16	44	6	76	56
BD-M	48	0	0	48	0	96	0	0	48	0	96	25	20	3	70	26	25	20	3	70	26	10	32	6	52	44
C-F	67	0	8	59	8	126	0	2	65	2	132	40	25	2	105	29	39	22	6	100	34	29	33	5	91	43
BD-F	40	0	1	39	1	79	0	1	39	1	79	20	17	3	57	23	19	18	3	56	24	7	21	12	35	45
GU	69	0	1	68	1	137	0	1	68	1	137	37	26	6	100	38	35	28	6	98	40	13	42	14	68	70
EN	37	0	1	36	1	73	0	1	36	1	73	20	16	1	56	18	19	17	1	55	19	5	22	10	32	42
PL	55	0	1	54	1	109	0	1	54	1	109	27	23	5	77	33	26	24	5	76	34	12	33	10	57	53
PPT	36	0	0	36	0	72	0	0	36	0	72	23	13	0	59	13	22	14	0	58	14	2	26	8	30	42
OI	41	0	1	40	2	80	0	1	40	2	80	19	18	4	56	26	20	17	4	57	25	9	23	9	41	41
A	53	0	1	52	1	105	0	1	52	1	105	24	23	6	71	35	22	25	6	69	37	10	31	12	51	55

C-M: Control male, C-F: Control female, BD-M: BD male, BD-F: BD female GU: Genital ulcers, EN: Erythema nodosum, PL: Papulopustular lesions, PPT: Positive pathergy test, OI: Ocular involvement, A: Arthritis

Supp Table S4. The distribution of *IL-23R* gene polymorphisms in gender and clinical findings of BD patients

Sex	n	rs1004819					rs7517847					rs7530511					rs10489629					rs10889677				
		GG	GT	TT	G+	T-	GG	GT	TT	G-	T+	CC	CT	TT	C+	T-	AA	AG	GG	A+	G-	AA	AC	CC	A-	C+
C-M	66	24	30	24	78	54	8	32	26	48	84	30	33	3	93	39	25	34	7	84	48	9	44	13	62	70
BD-M	48	11	21	16	43	53	5	20	23	30	66	20	23	5	63	23	21	19	8	61	35	9	32	7	50	46
C-F	67	8	51	8	67	67	8	34	25	50	84	27	40	0	94	40	29	34	4	92	42	8	53	6	69	65
BD-F	40	14	19	7	47	33	5	18	17	28	52	16	19	5	51	29	8	20	12	36	44	5	24	11	34	46
GU	69	18	33	18	69	69	5	30	34	40	98	28	35	6	91	47	23	31	15	77	61	12	42	15	66	72
EN	37	13	16	8	42	32	3	15	19	21	53	15	16	6	46	28	11	17	9	39	35	3	23	11	29	45
PL	55	19	23	13	61	49	7	24	24	38	72	22	26	7	70	40	19	23	12	61	49	9	33	13	51	59
PPT	36	11	15	10	37	35	5	17	14	27	45	18	13	5	49	23	10	16	10	36	36	6	22	8	34	38
OI	41	15	16	10	46	36	9	14	18	32	50	22	14	5	58	24	11	17	13	39	43	6	23	12	35	47
A	53	20	19	14	59	47	7	19	27	33	73	25	23	5	73	33	19	19	15	57	49	10	30	13	50	56

C-M: Control male, C-F: Control female, BD-M: BD male, BD-F: BD female GU: Genital ulcers, EN: Erythema nodosum, PL: Papulopustular lesions, PPT: Positive pathergy test, OI: Ocular involvement, A: Arthritis

Supp Table S5. Haplotypes of *IL-12B* and *IL-23R* together

	Haplotypes (IL-12B-IL23R)	BH	HCs		Haplotypes	BH	HCs
**1**	GAAGG-GTCAC	7.95 ± 2.05	11.28 ± 1.94	**51**	AAAGG-GGTAC	0.57 ± 0.57	-
**2**	AAAGG-TGTGA	7.39 ± 1.98	6.01 ± 1.46	**52**	GCCGG-TTTAA	0.57 ± 0.57	-
**3**	GCCGG-GTCAC	6.82 ± 1.90	6.01 ± 1.46	**53**	GCCAT-TTCAA	0.57 ± 0.57	-
**4**	AAAGG-TTCAA	5.11 ± 1.67	4.89 ± 1.32	**54**	AAAGG-GGCGA	0.57 ± 0.57	1.50 ± 0.75
**5**	AAAGG-TTTAA	3.98 ± 1.48	-	**55**	AAAGG-GGTGC	0.57 ± 0.57	-
**6**	GAAGG-TTCAA	3.98 ± 1.48	2.26 ± 0.91	**56**	GCCGG-GTTAC	0.57 ± 0.57	-
**7**	AAAGG-TGCGA	3.98 ± 1.48	6.01 ± 1.46	**57**	ACCGG-GTTAC	0.57 ± 0.57	-
**8**	ACCGG-GTCAC	3.41 ± 1.37	4.13 ± 1.22	**58**	AAAGG-TTTAC	0.57 ± 0.57	0.38 ± 0.38
**9**	GCCGG-GTCGC	2.84 ± 1.26	-	**59**	AAAGG- TTTAA		5.26 ± 1.37
**10**	AAAGG-GGTGA	2.84 ± 1.26	2.63 0.98	**60**	AACGG-GTCAC		1.50 ± 0.75
**11**	AAAGG-GTCAC	2.84 ± 1.26	4.51 ± 1.28	**61**	AAAGG-GTTAA		1.13 ± 0.65
**12**	GCCGG-TTCAC	2.27 ± 1.13	-	**62**	GAAGT-GTCAC		1.13 ± 0.65
**13**	GAAGG-TGTGA	2.27 ± 1.13	-	**63**	GCAGG- GTCAC		0.75 ± 0.53
**14**	AAAGG-TTCGA	2.27 ± 1.13	2.26 ± 0.91	**64**	GACGG-GTCAC		0.75 ± 0.53
**15**	GAAGG-TTTGA	2.27 ± 1.13	-	**65**	GCCGG-GGCGC		0.75 ± 0.53
**16**	AAAGG-TGTAA	2.27 ± 1.13	0.38 ± 0.38	**66**	ACCGG-GGCAC		0.75 ± 0.53
**17**	GAAGG-GGCGC	2.27 ± 1.13	1.50 ± 0.75	**67**	GAAGG-TGTAA		0.75 ± 0.53
**18**	GAAGG-GTTGA	1.71 ± 0.98	-	**68**	GCCGT-TTCAA		0.75 ± 0.53
**19**	GAAGG-GTCGC	1.71 ± 0.98	0.38 ± 0.38	**69**	GCCGT-GGCGC		0.75 ± 0.53
**20**	AAAGG-GTCGC	1.71 ± 0.98	2.63 ± 0.98	**70**	ACAGG-TGTGA		0.75 ± 0.53
**21**	AAAGG-GGCGC	1.71 ± 0.98	0.75 ± 0.53	**71**	ACAGG-GTCAC		0.75 ± 0.53
**22**	GAAGG-TTCAC	1.71 ± 0.98	0.75 ± 0.53	**72**	ACCGG-GTCAA		0.75 ± 0.53
**23**	GCCGG-TTCAA	1.71 ± 0.98	0.75 ± 0.53	**73**	ACCGG-TGTGA		0.38 ± 0.38
**24**	GAAGG-TTCGC	1.14 ± 0.80		**74**	GCAGG-GTTAC		0.38 ± 0.38
**25**	ACCGG-GGCGC	1.14 ± 0.80	0.38 ± 0.38	**75**	GCCGG- TTCAC		0.38 ± 0.38
**26**	ACCGG-GGTGA	1.14 ± 0.80		**76**	AACGG-TGTGA		0.38 ± 0.38
**27**	AAAGG-TTTGA	1.14 ± 0.80	1.13 ± 0.65	**77**	GCCGT-GTCAC		0.38 ± 0.38
**28**	ACCGG-TTCAA	1.14 ± 0.80	1.13 ± 0.65	**78**	GAAGT-TTCAA		0.38 ± 0.38
**29**	GAAGG-GTTAA	1.14 ± 0.80	-	**79**	GAAGG-GGCAA		0.38 ± 0.38
**30**	GAAGG-GTTAC	1.14 ± 0.80	0.38 ± 0.38	**80**	ACCGT-TTCAC		0.38 ± 0.38
**31**	GAAGG-GGCAC	1.14 ± 0.80	0.38 ± 0.38	**81**	GAAGT-GTCGC		0.38 ± 0.38
**32**	GCCGG-GGCAC	1.14 ± 0.80	1.50 ± 0.75	**82**	GAAGT-GGCGC		0.38 ± 0.38
**33**	GAAGG-TTCGA	0.57 ± 0.57	0.38 ± 0.38	**83**	GACGG-GTCGC		0.38 ± 0.38
**34**	GAAGG-TGCGA	0.57 ± 0.57	1.13 ± 0.65	**84**	GACGG-GTTAC		0.38 ± 0.38
**35**	AAAGG-TGCAA	0.57 ± 0.57	0.75 ± 0.53	**85**	AAAGG-GGCAC		0.38 ± 0.38
**36**	GAAGG-GTCAA	0.57 ± 0.57	1.13 ± 0.65	**86**	AAAGG-GGCAA		0.38 ± 0.38
**37**	GACGG-TTTGC	0.57 ± 0.57	-	**87**	ACAAT-GTCAC		0.38 ± 0.38
**38**	GCCGG-TTCGC	0.57 ± 0.57	-	**88**	ACCGG-TTCAC		0.38 ± 0.38
**39**	AAAGG-TGTGC	0.57 ± 0.57	0.75 ± 0.53	**89**	ACAGG-TTCGA		0.38 ± 0.38
**40**	AAAGG-GTTGC	0.57 ± 0.57	0.75 ± 0.53	**90**	AAAGG-GTCGA		0.38 ± 0.38
**41**	GCAGG-GTTAA	0.57 ± 0.57	-	**91**	AACGG-TTCAA		0.38 ± 0.38
**42**	ACCGG-TTTAA	0.57 ± 0.57	0.38 ± 0.38	**92**	AAAGT-GTCGC		0.38 ± 0.38
**43**	GAAGG-GGCGA	0.57 ± 0.57	-	**93**	AAAGG-GGTAA		0.38 ± 0.38
**44**	AAAGG-TGCGC	0.57 ± 0.57	0.38 ± 0.38	**94**	AAAGG-GTTAC		0.38 ± 0.38
**45**	ACCGG-GTCGG	0.57 ± 0.57	-	**95**	GAAAT-GTCAC		0.38 ± 0.38
**46**	GCAGG-GTCGC	0.57 ± 0.57	-	**96**	GCAGG-TTCAC		0.38 ± 0.38
**47**	ACCGG-GTTGC	0.57 ± 0.57	-	**97**	ACAGG-TTTAA		0.38 ± 0.38
**48**	ACCGG-GTTAA	0.57 ± 0.57	-	**98**	ACAGG-TGTAA		0.38 ± 0.38
**49**	AAAGG-TTCAC	0.57 ± 0.57	0.38 ± 0.38	**99**	AAAAT-GTCAC		0.38 ± 0.38
**50**	GCCGG-GGTAC	0.57 ± 0.57	-	**100**	ACAGG-GGTAA		0.38 ± 0.38

Supp Table S6. Clinical findings and SNP associations of *IL-12B* and *IL-23R*

	SNP	Genotype / Allele	OR	95%CI	*p*
**GENDER**					
**IL-12B**					
BD-F / HC-F	rs2082412	AA	0.2780	0.1077- 0.7173	0.0081
BD-F / HC-F	rs2082412	GG	5.3143	1.7086 - 16.529	0.0039
BD-F / HC-F	rs2082412	A	0.3675	0.2075 - 0.6509	0.0006
BD-F / HC-F	rs2082412	G	2.7209	1.5364 - 4.8189	0.0006
**IL-23R**					
BD-F / HC-F	rs7530511	TT	20.9155	1.124 - 389.156	0.0415
BD-F / HC-F	rs10489629	GG	6.7500	2.0007 - 22.772	0.0021
BD-F / HC-F	rs10489629	AA	0.3276	0.1314 - 0.8164	0.0166
BD-F / HC-F	rs10489629	G	2.6772	1.5109 - 4.7438	0.0007
BD-F / HC-F	rs10489629	A	0.3735	0.2108 - 0.6618	0.0007
BD-M /HC-M	rs1004819	T	1.7804	1.0466 - 3.0285	0.0333
BD-M /HC-M	rs1004819	G	0.5617	0.3302 - 0.9554	0.0333
**CLINICAL FINDINGS**					
**IL-12B**					
P+ / P-	rs2082412	AA	0.1451	0.0309 - 0.6817	0.0145
P+ / P-	rs3212227	AA	2.4126	1.0060 - 5.7861	0.0485
P+ / P-	rs3212227	A	2.4027	1.1651 - 4.9548	0.0176
P+ / P-	rs3212227	C	0.4162	0.2018 - 0.8583	0.0176
P+ / P-	rs3213113	A	2.1933	1.0785 - 4.4603	0.0301
P+ / P-	rs3213113	C	0.4559	0.2242 - 0.9272	0.0301
A+ / -A-	rs3213113	A	0.4253	0.2065 - 0.8762	0.0204
A+ / -A-	rs3213113	C	2.3512	1.1413 - 4.8438	0.0204
V+ / V-	rs3213119	GG	0.0311	0.0012 - 0.8311	0.0384
V+ / V-	rs3213119	GT	32.2000	1.2032 - 861.73	0.0384
V+ / V-	rs3213119	G	0.0322	0.0013 - 0.8243	0.0378
V+ / V-	rs3213119	T	31.0645	1.2132 - 795.45	0.0378
V+ / V-	rs3213120	GG	0.0311	0.0012 - 0.8311	0.0384
V+ / V-	rs3213120	AG	32.2000	1.2032 - 861.73	0.0384
V+ / V-	rs3213120	G	0.0322	0.0013 - 0.8243	0.0378
V+ / V-	rs3213120	A	31.0645	1.2132 - 795.45	0.0378
V+ / V-	rs3213113	AC	11.0645	1.2978 - 94.330	0.0279
		**IL-23R**			
E+ /E-	rs10889677	AA	0.2406	0.0613 - 0.9449	0.0412
A+ /A-	rs1004819	GT	0.3725	0.1547 - 0.8972	0.0277
A+ /A-	rs1004819	GG	3.6364	1.2133 - 10.898	0.0212
OL+ /OL-	rs7517847	GG	12.9375	1.5611 - 107.22	0.0177
OL+ /OL-	rs7530511	CT	0.3519	0.1475 - 0.8394	0.0185
OL+ /OL-	rs7530511	CC	2.7293	1.1365 - 6.5548	0.0247
GL+ / GL-	rs7517847	GG	0.2188	0.0557 - 0.8590	0.0294
GL+ / GL-	rs7517847	G	0.4535	0.2174 - 0.9461	0.0351
GL+ / GL-	rs7517847	T	2.2050	1.0569 - 4.6001	0.0351

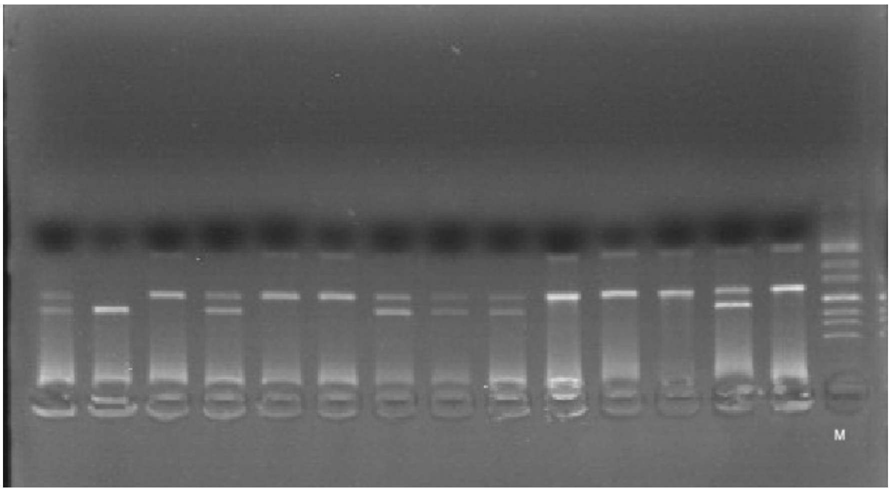

Supp Figure S1: PCR-RFLP results of IL-23, rs3213113 on 4% agarose gel electrophoresis. *Marker (M): Ultra Low Range DNA Ladder, PCR product: 300bp, homozygous genotype (+/+): 204bp, 96bp, heterozygous genotype (+/-): 300bp, 204bp, 96bp.*

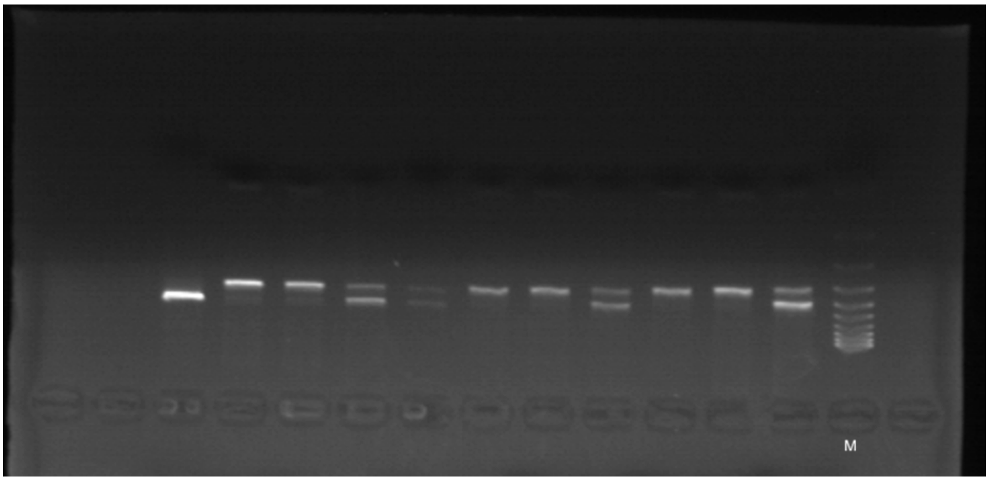

Supp Figure S2: PCR-RFLP results of IL-23R, rs7517847 on 2% agarose gel electrophoresis. *Marker: 100bp DNA ladder, PCR product: 530bp, homozygous genotype (+/+): 410bp, 91bp, 29bp, heterozygous genotype (+/-): 530bp, 410bp, 91bp, 29bp.*
